# Proactive Irrigation Timing Decision-Making for Greenhouse Tomatoes via STL-LSTM Deep Learning and Plant–Soil Dual-Threshold Sensing

**DOI:** 10.3390/s26102981

**Published:** 2026-05-09

**Authors:** Wei Zhou, Zhenglin Li, Yuande Dong, Longjie Li, Shuo Liu

**Affiliations:** 1 College of Mechanical and Electrical Engineering, Xinjiang Agricultural University, Urumqi 830052, China; 13779268278@163.com (Z.L.); dydjdxy@xjau.edu.cn (Y.D.); lidelongjie@163.com (L.L.); 18146404138@163.com (S.L.); 2 Key Laboratory of Xinjiang Intelligent Agricultural Equipment, Urumqi 830052, China

**Keywords:** tomato, stem diameter, STL-LSTM, irrigation scheduling, water stress, solar greenhouse

## Abstract

**Highlights:**

By first establishing a dual-threshold irrigation trigger mechanism based on Maximum Daily Shrinkage (MDS > 70 μm) and Soil Volumetric Water Content (VWC ≤ 17%), this study identified the critical physiological boundaries of water deficit in tomatoes. On this basis, a hybrid STL-LSTM model was developed to achieve high-precision 24-h forecasting of stem diameter (R^2^ = 0.9184). This integrated approach facilitates a paradigm shift from passive soil moisture monitoring to proactive, plant-centric precision irrigation management, providing a scalable technical solution to optimize water-use efficiency and ensure crop health in arid-zone greenhouse agriculture.

**What are the main findings?**

- A robust dual-threshold irrigation trigger was established, identifying that a Maximum Daily Shrinkage (MDS) exceeding 70 μm combined with soil moisture below 17% signifies critical water stress.
- The hybrid STL-LSTM model achieved high-precision (R^2^ = 0.9184) 24-h forecasting of tomato stem diameter by effectively decomposing non-stationary physiological signals into seasonal and trend components.

**What are the implications of the main findings?**

- Integrating plant-based physiological sensors with deep learning enables a paradigm shift from traditional, passive soil moisture monitoring to proactive, plant-centric precision irrigation management.
- The proposed framework provides a scalable technical solution for optimizing water-use efficiency and ensuring crop health in arid-zone greenhouse agriculture, mitigating risks of empirical scheduling errors.

**Abstract:**

Traditional irrigation management for tomatoes in solar greenhouses relies heavily on empirical manual experience and single soil moisture indicators, often leading to irrigation scheduling that lacks crop-specific physiological evidence and results in suboptimal water-use efficiency. To address these challenges, this study developed an intelligent, plant-centric irrigation decision-making framework for greenhouse tomatoes in the arid region of Xinjiang. Central to this framework is the precise identification of irrigation timing—the most critical first step and a fundamental prerequisite for achieving true on-demand irrigation. By monitoring the high-frequency dynamics of stem diameter (SD) and integrating soil moisture data, the physiological responsiveness of tomatoes to water stress was systematically analyzed. A hybrid predictive model, STL-LSTM, was constructed by coupling Seasonal-Trend decomposition using Loess (STL) with Long Short-Term Memory (LSTM) networks to forecast 24-h SD trends. Furthermore, an innovative dual-threshold irrigation mechanism was established, utilizing a physiological trigger (Maximum Daily Shrinkage, MDS > 70 μm) and a soil moisture constraint (Volumetric Water Content, VWC ≤ 17%). Results demonstrated that tomato SD exhibited distinct diurnal rhythms, with MDS and Daily Increment (DI) identified as highly sensitive indicators of plant water status. The proposed STL-LSTM model achieved superior predictive performance during the peak fruiting stage, with a coefficient of determination (R^2^) of 0.9184, representing an improvement of 14.8% and 27.56% over standalone LSTM and ARIMA models, respectively. The validation of the dual-threshold mechanism confirms its ability to balance real-time crop water demand with conservation requirements, effectively mitigating the risks of premature or delayed irrigation inherent in traditional methods. This research provides scientific rationale and technical support for the transition of greenhouse agriculture in arid regions towards precision irrigation and optimised water resource management.

## 1. Introduction

Tomatoes rank among the primary cultivated varieties in China’s protected-culture vegetable sector, characterised by ease of cultivation, substantial market demand, and high economic returns. Both their cultivation area and yield lead the nation’s protected agriculture sector [1,2]. Irrigation, as a core component in regulating greenhouse tomato growth, significantly impacts development through its scientific precision. Water requirements during the fruiting and peak fruiting stages account for 60–70% of the entire growth period. Water deficits during this phase can reduce fruit set by 15–20%, decrease individual fruit weight by 10–15%, and lower soluble sugar content by 8–12% [3]. However, under the ridge-based plastic-covered drip irrigation cultivation model widely adopted in northern solar greenhouses, current irrigation regimes have yet to establish water regulation strategies precisely aligned with crop water demand patterns. Consequently, conducting irrigation decision-making research for tomatoes based on precise water demand thresholds within this model holds significant importance for optimising water management, safeguarding yield and quality, and enhancing water resource utilisation efficiency.

In recent years, research by scholars both domestically and internationally in the field of precision irrigation has primarily focused on the following areas: irrigation regimes based on soil moisture [4,5], which primarily utilise multiple sensors and parameter modelling techniques to predict soil moisture. By comparing water content across different plant growth stages and integrating IoT applications into agriculture, these methods aim to reduce water consumption and enhance yields. However, a drawback lies in their neglect of the relationship between environmental factors and irrigation. Conversely, plant physiological changes represent responses to the water status within the plant’s own tissues rather than to soil moisture content [6], thus failing to accurately reflect the crop’s intrinsic moisture status. Simulation studies employing crop irrigation models [7,8,9] simulate crop irrigation volumes over fixed periods based on multi-year data. Their drawbacks include relatively complex calculations, limited applicability, and lack of timeliness [10].

Numerous methods exist for monitoring and diagnosing crop water status. From a plant physiological perspective, short-term dynamic micro-changes in the volume of plant organs (such as stems, leaves, and fruits) are closely linked to their internal water conditions. When roots absorb sufficient water, stems expand; when water is deficient, stems contract. This phenomenon enables the use of stem diameter micro-change indicators to diagnose crop water deficiency and guide irrigation [11,12]. Systematic research into monitoring crop water status via stem diameter variation commenced in the 1960s. Early findings demonstrated a strong correlation between the degree of stem diameter contraction and expansion and the plant’s internal water status, a phenomenon observed across diverse crops including cotton, soybean, cabbage, tomato, and fruit trees [13,14]. Stem diameter metrics suitable for automated irrigation control were proposed, including the maximum daily shrinkage (MDS), maximum/minimum daily increment (DI), and the time required for complete recovery of the maximum daily stem diameter within a 24-h cycle (RT) [15,16]. The stem diameter variation method is characterised by its simplicity, non-destructive nature, and capability for continuous automated monitoring. Consequently, it has attracted considerable research attention and is increasingly applied to monitor and diagnose crop water status [17,18]. Blanco et al. [19] proposed employing maximum daily shrinkage (MDS) as a diagnostic indicator for crop water status and irrigation guidance, initiating irrigation when the plant stem’s MDS reaches a predetermined threshold. Zhang Jiyang et al. [20,21] observed that stem diameter variation effectively reflects cotton water status. However, stem diameter changes are influenced by both environmental factors and crop developmental characteristics, necessitating different indicators at distinct growth stages. Wang Xiaosen et al. [22] investigated the mechanisms and patterns of stem diameter variation in greenhouse tomatoes, the impact of environmental factors on stem diameter changes, and methods to mitigate meteorological interference in measured MDS values. Li Hui et al. [23] investigated the patterns of stem diameter variation in summer maize, observing that the daily maximum stem diameter decreased with reduced soil moisture content, exhibiting a linear correlation between the two. Based on this relationship, subtle changes in stem diameter can be utilised to diagnose crop water deficiency.

In recent years, various modeling approaches have been developed to predict stem diameter variations (SDV). Early studies primarily relied on multiple linear regression to link environmental factors with SDV, but these models often struggled to capture the non-linear physiological responses under complex conditions [24] (Wu et al., 2021). With the advent of machine learning, algorithms such as Random Forest (RF) and Multi-Layer Perceptron (MLP) have been applied to improve prediction accuracy [25] (Johansen et al., 2020); however, these methods frequently overlook the inherent temporal dependency and sequential logic of plant growth. To address this, deep learning architectures like Long Short-Term Memory (LSTM) [26] (An et al., 2022) and Gated Recurrent Unit (GRU) [27] (Li et al., 2023) have shown promise, achieving R^2^ values between 0.87 and 0.90.

Although existing research has extensively demonstrated that changes in stem diameter serve as an effective diagnostic indicator of crop water deficit, with most studies focusing on analysing its diagnostic feasibility and influencing factors, research directly applying this to guide precision irrigation decisions remains relatively scarce.

This study focuses on the requirements for precision irrigation in solar greenhouse tomato production. Using tomato plants during the peak fruiting stage as experimental subjects, we monitored the dynamic response of Maximum Daily Stem Shrinkage (MDS) under gradient water stress. Crucially, while MDS is highly sensitive to plant water status, its practical application is often confounded by atmospheric noise; high evaporative demand can trigger significant stem contraction even when soil moisture is non-limiting, leading to premature or “false-positive” irrigation triggers in conventional single-indicator systems.

To address these limitations, we developed a stem diameter prediction model and proposed the ‘Plant-Soil Dual-Threshold Sensing’ framework. Unlike traditional methods, this framework operates on a ‘double-insurance’ logic: it utilizes the predicted MDS as a primary biological trigger to sense the plant’s immediate water demand, which is then cross-verified by the physical constraints of Soil Volumetric Water Content (VWC). Irrigation is initiated if and only if both physiological and soil thresholds are satisfied. This synergistic sensing approach effectively filters out transient atmospheric-induced fluctuations, overcoming the inherent blind spots of single-indicator monitoring. It provides a robust theoretical foundation for precision irrigation.

## 2. Materials and Methods

### 2.1. Overview of the Experimental Greenhouse and Varieties Tested

**Figure 1:**
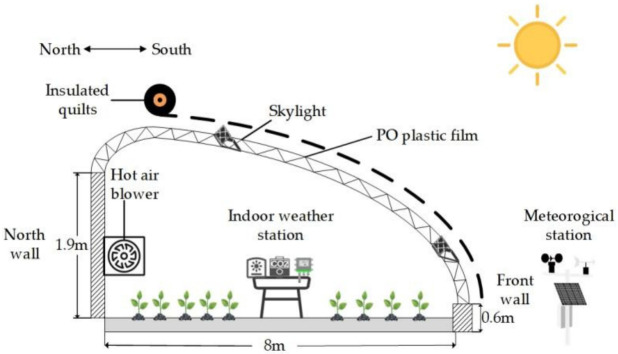
Cross-section of the test greenhouse.

The trials were conducted at the Sanping Teaching and Practice Base of Xinjiang Agricultural University (43.92° N, 87.35° E) from 1 September 2024 to 20 December 2024 and from 20 March 2025 to 20 July 2025. The test solar greenhouse was oriented north–south, measuring 44 m in east–west length with an 8 m span. It featured a ridge height of 2.6 m, a rear wall height of 1.9 m, a front wall height of 0.6 m, and lacked a rear slope. The front roof surface employed PO plastic film, externally covered with thermal blankets. The experimental greenhouse structure is as shown in Figure 1.

The experimental material comprised the indeterminate tomato cultivar ‘Provence’ (*Solanum lycopersicum* L.). Two crop cycles were established: the first cycle commenced on 1 September 2024, employing a north–south ridge cultivation with plastic mulching within a solar greenhouse. Plant spacing was 37 cm × 30 cm, with emphasis on acquiring physiological and environmental characteristic data under actual production conditions; The second round of planting was carried out on 20 March 2025 using the pot-cultivation method, with the aim of precisely calibrating physiological response thresholds. The tomatoes were planted in plastic pots measuring 28 cm in diameter and 30 cm in height. Each pot was filled with 10 kg of air-dried soil (sifted through a 2 mm sieve, with an initial moisture content of 3.8%), to a depth of 21 cm. Real-time monitoring of pot soil weight changes was achieved via high-frequency weighing, enabling precise quantitative control of water gradients.

The irrigation treatments were based on Field Capacity (FC), which represents the maximum amount of water the soil can retain after excess gravitational water has drained away. The experimental soil was a sandy loam. Prior to the experiment, the FC was determined using the standard cutting ring method (100 cm^3^ volume, n = 5). Undisturbed soil cores were saturated with water for 24 h and then allowed to drain freely for 48 h on a sand bed until the weight stabilized. The samples were then oven-dried at 105 °C to determine the dry soil mass and bulk density. To maintain consistency between the soil physical properties and the experimental conditions, the pots were filled with sieved soil from the same source and packed in layers to match the pre-determined field bulk density. The measured FC was 26.0% (volumetric water content, cm^3^/cm^3^), which served as the baseline for calculating irrigation thresholds.

### 2.2. Experimental Design and Moisture Gradient Control

In the first crop trial, water management referenced the local conventional irrigation regime. Soil moisture sensors monitored volumetric water content in the 0–20 cm soil layer in real time, providing training and validation datasets for the STL-LSTM model at actual production scale.

In the second crop trial, the following water treatments were established to precisely calibrate physiological response thresholds in tomatoes:

Constant Gradients: Three treatments were established: T1 (control, 80–100% FC), T2 (mild stress, 60–80% FC), and T3 (moderate stress, 40–60% FC). Water was replenished daily at 08:00 and 20:00 using electronic weighing methods to maintain soil moisture within target ranges.

Progressive Drought (PD): Select uniformly growing plants and cease all irrigation during peak fruiting until permanent wilting point is reached. This treatment yields a continuous physiological response curve from fully hydrated to severely drought-stressed conditions.

Each treatment group comprised three replicates (five plants per replicate). Utilising continuous monitoring data from the PD group, combined with steady-state performance from T1–T3, the critical point where the maximum daily stem shrinkage (MDS) exhibited a non-linear shift was identified as the physiological threshold for early warning.

MDS serves as a high-sensitivity ‘early warning’ signal for plant water deficit. It reflects the dynamic dehydration of stem storage tissues (such as phloem and cambium) when transpiration exceeds root water uptake. Unlike soil moisture which represents water supply, MDS represents the plant’s actual water demand. We employed MDS to identify the ‘Critical Transition’ point—where the plant shifts from a state of luxury growth to one of water-limited growth. This provides a more biologically relevant trigger for irrigation than soil moisture alone, as it directly mirrors the plant’s internal hydraulic status.

**Figure 2:**
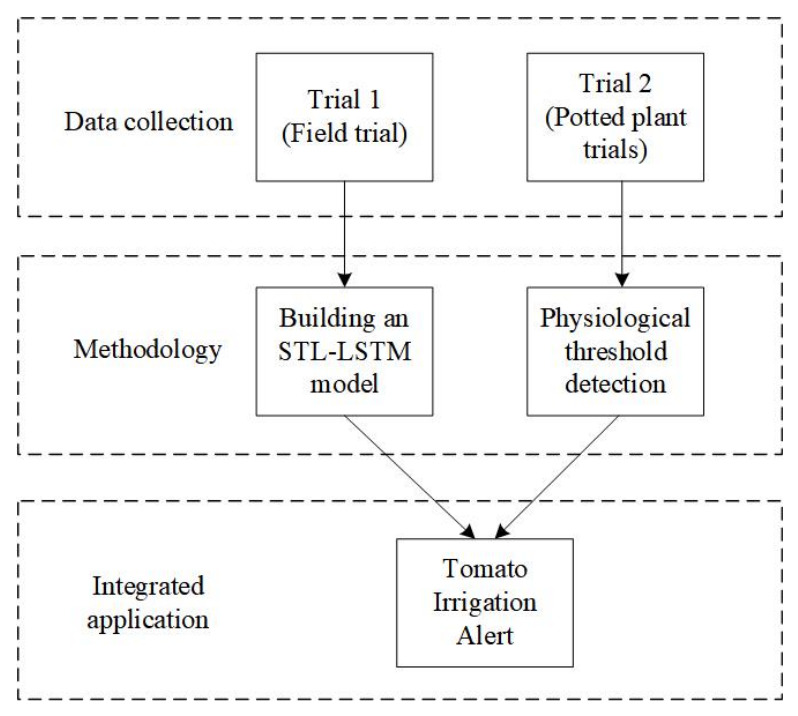
Roadmap for Group Experimentation.

To facilitate a holistic understanding of the experimental design and data analysis, the overall research framework is illustrated in Figure 2. This study integrates a field-scale trial (Trial 1) for developing the STL-LSTM soil moisture prediction model and a controlled pot-scale trial (Trial 2) for identifying tomato physiological response thresholds. The synergy of these two components enables a comprehensive approach to precision irrigation early warning.

### 2.3. Sensor Integration and Data Acquisition

The experimental data acquisition platform utilises the PH automatic weather station manufactured by Wuhan New HP Technology Co., Ltd. (Wuhan, China), the air temperature and humidity meter produced by Jiangsu Jingchuang Company (Xuzhou, China), and meteorological instruments manufactured by Zhejiang Top Cloud Agricultural Formula (Hangzhou, China). Parameters collected include outdoor temperature, outdoor humidity, outdoor light intensity, outdoor wind speed, indoor CO_2_ concentration, indoor temperature, indoor humidity, indoor light intensity, and tomato stem diameter. Continuous monitoring of soil volumetric water content (VWC) was carried out using high-precision soil moisture sensors (NL-GPRS-I), (New HP, Wuhan, China). In each treatment, three sensors were installed horizontally at a depth of 15 cm at the centre of the root zone to ensure representative tracking of moisture dynamics. These sensors, based on Time Domain Reflectometry (TDR) technology, have a measurement accuracy of ±1%. All sensors were cross-calibrated against weighing data obtained during the FC determination process to ensure the objectivity and comparability of all moisture content data series.

**Figure 3:**
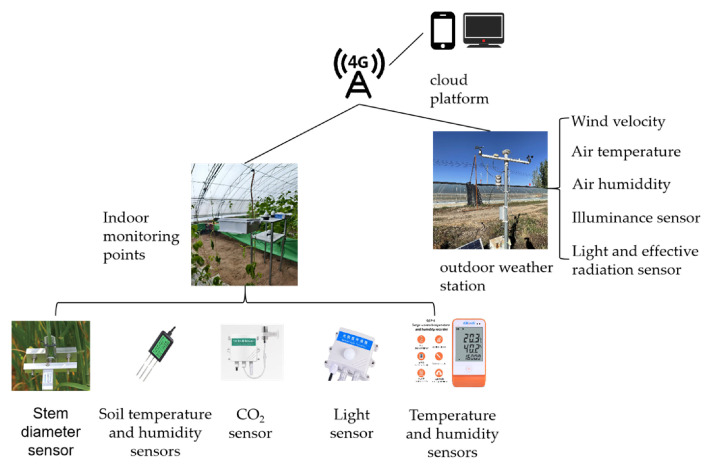
Schematic diagram of environmental data acquisition system. Note: The non-English text and unclear partial details on the sensor and device photos in this schematic are only commercial branding and irrelevant auxiliary information, which do not affect the scientific understanding of the overall system structure and experimental principle.

**Figure 4:**
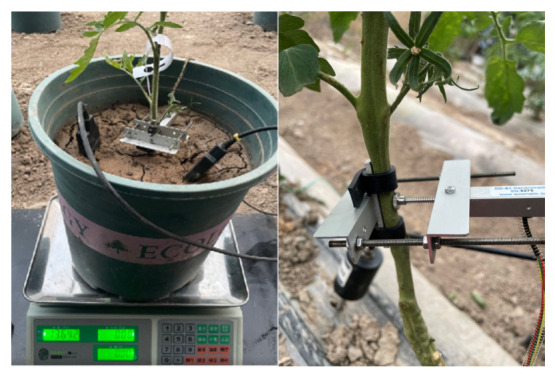
Potted tomato weight, stem diameter, and soil moisture dynamic monitoring. Note: Non-English text on the scale includes brand information and operation labels, which are standard device markings and irrelevant to the experiment.

The structure of the automated greenhouse environmental monitoring system is illustrated in Figure 3, with tomato stem diameter monitoring depicted in Figure 4. Specific parameters are detailed in Table 1.

Throughout both crop trials, Changes in tomato stem diameter were continuously monitored non-destructively using a high-precision linear displacement sensor (Model: DD-S1, Manufacturer: ECOMATIK GmbH, Dachau, Germany). The sensor was mounted midway along the main stem of representative plants (approximately 10–20 cm above ground level) using a custom stainless steel bracket. This ensured the sensor probe maintained perpendicular and close contact with the stem surface while preventing mechanical damage to plant tissue. To ensure experimental replication, three stem diameter sensors and three soil moisture sensors were deployed per treatment during each trial (one sensor per representative plant in each of the three replicates). Monitoring data for both seasons was automatically recorded by a data logger (Model: DL-18), set to sample and store data every 30 min, thereby capturing daily dynamic variations in stem diameter.

### 2.4. Data Processing and Feature Extraction

This study continuously collected environmental and physiological data during the tomato fruit-setting period at a sampling frequency of 30 min. To enhance the robustness of the inputs for the predictive model, the raw measurement data from the three sensors in each treatment were aggregated by calculating the arithmetic mean at each time step. This aggregation strategy was employed to generate a representative physiological signal for each treatment group and to minimize the influence of individual plant growth variability (noise).

After aggregation, a total of 2928 time-series data sets were accumulated. Each data set encompasses 12 feature dimensions (including both environmental parameters and aggregated physiological indices). To address noise and anomalous fluctuations within the raw sensor sequences, the data cleaning and normalisation process was as follows:

(1) Outlier handling and missing value imputation: Boxplots were employed to identify and remove outliers. To avoid spurious long-range interpolation, a tiered strategy was adopted for missing value restoration: sparse missing points were filled using mean imputation or linear interpolation; for larger missing spans, analogous historical data from neighbouring time periods with similar meteorological conditions (e.g., irradiance, temperature, humidity) were selected for analogical filling.
(2) Dimensional Normalisation: To eliminate order-of-magnitude differences between environmental factors and enhance model convergence speed, input environmental factors undergo normalisation. Data is mapped to the 0–1 range. For input variables X1, X2, X3… XN, the normalisation formula is calculated as follows:

(1) $$X=\frac{X_{i}-Min(X_{c})}{Max(X_{c})-Min(X_{c})}$$

In the formula, Max and Min denote the maximum and minimum values within the training set, X represents the normalised data, and c is a positive integer.

**Figure 5:**
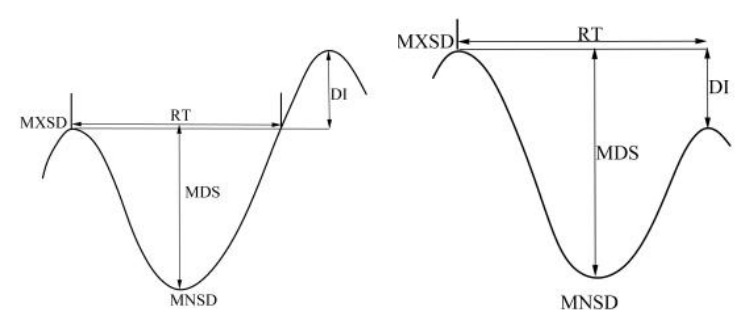
Dynamic diagram of tomato stem diameter change.

This study extracted stem diameter variation metrics for analysis, the daily maximum (MXSD) and daily minimum (MNSD) denote the highest and lowest stem diameters recorded within a single day, respectively. The daily maximum shrinkage (MDS) represents the difference between the daily maximum and minimum values. The daily increment (DI) is calculated as the difference between the current day’s MXSD and the previous day’s MXSD. RT denotes the time required for recovery on the current day [28,29,30]. As illustrated in Figure 5, this represents scenarios where stem diameter recovers with growth within 24 h and where incomplete recovery occurs, with RT calculated over a 24-h period.

### 2.5. Phyto-Sensing-Based Calibration Protocol for Tomato Water Stress Thresholds

By comparing the physiological responses of plants under fully irrigated (T1) and progressively drought-stressed (PD) treatments, the threshold for determining water stress in tomatoes was established. To ensure the applicability of the results to production environments, the calibration trials were conducted using potted tomatoes placed directly within the production greenhouse, thereby matching the atmospheric evaporative demand (e.g., VPD and radiation) of real-world systems. Continuous diameter change curves were obtained using DD-S1 sensors mounted on stems, with maximum daily shrinkage (MDS), daily increment (DI), and recovery time (RT) extracted as core evaluation metrics. The calibration process was based on comparative trials between the fully irrigated group (T1) and the progressively dried group (PD). The use of pot-based cultivation allowed for a more precise and uniform control of soil water depletion compared to open-soil systems, which is essential for accurately capturing the physiological “tipping point.” Continuous stem diameter variation signals were obtained using high-precision displacement sensors, with crop growth recovery capacity serving as the benchmark indicator for determining water status.

Critical threshold determination: The identification of growth arrest is grounded in the “zero-growth” physiological framework, which distinguishes irreversible structural expansion from reversible water-related fluctuations [31] (Zweifel, 2016). According to this concept, stem growth occurs only when nocturnal turgor pressure exceeds a specific threshold, allowing the stem diameter to surpass its previous maximum. To ensure the objectivity of the threshold, a systematic quantitative protocol was employed instead of empirical observation. For each replicate in the PD group, the specific day on which the plant failed to restore its maximum night-time stem diameter to the previous day’s level (RT = 24 h) and exhibited a non-positive daily increment (DI ≤ 0) was identified as the physiological tipping point.

The specific MDS value for the diurnal cycle corresponding to this growth-arrest day was then extracted for each individual plant. The final early-warning critical threshold was derived by calculating the arithmetic mean of these individual MDS values across all replicates. This state signifies that the nocturnal rehydration was insufficient to overcome the daytime water deficit and generate enough turgor to drive cell expansion [32]. While root volume and soil water dynamics may vary between pots and larger greenhouse systems, this MDS threshold—as a physiological proxy—reflects the plant’s integrated response to water imbalance, making it a robust and transferable indicator for predicting water deficit timing across different growing scales.

The critical MDS threshold derived from this calibration protocol serves as the primary biological trigger within the broader ‘Plant-Soil Dual-Threshold Sensing’ framework. To bridge the gap between plant physiological signals and practical soil management, the Soil Volumetric Water Content (VWC) corresponding to this physiological tipping point was simultaneously recorded as the physical threshold. This integration ensures that the calibrated MDS signal does not act in isolation; rather, it initiates a ‘double-insurance’ sensing logic where irrigation is only triggered when the biological demand (MDS) is cross-verified by physical soil water depletion (VWC). By coupling these two dimensions, the sensing framework effectively filters out transient environmental noise and ensures that irrigation precisely targets genuine, soil-induced water deficits.

## 3. Model Construction and Implementation

This study proposes a hybrid forecasting framework integrating STL (Seasonal-Trend Decomposition using Loess) decomposition with multi-feature Long Short-Term Memory (LSTM) networks. By deconstructing the complex dynamic characteristics of stem diameter sequences, the method separately models trend, seasonal, and residual components to capture both the long-term evolutionary patterns and short-term nonlinear fluctuations in crop growth.

To ensure the model captures the physiological response of stem dynamics to environmental fluctuations, four external variables were selected as input features: Air Temperature (T), Relative Humidity (H), Light Intensity (L), and Soil Moisture (SM).

**Figure 6:**
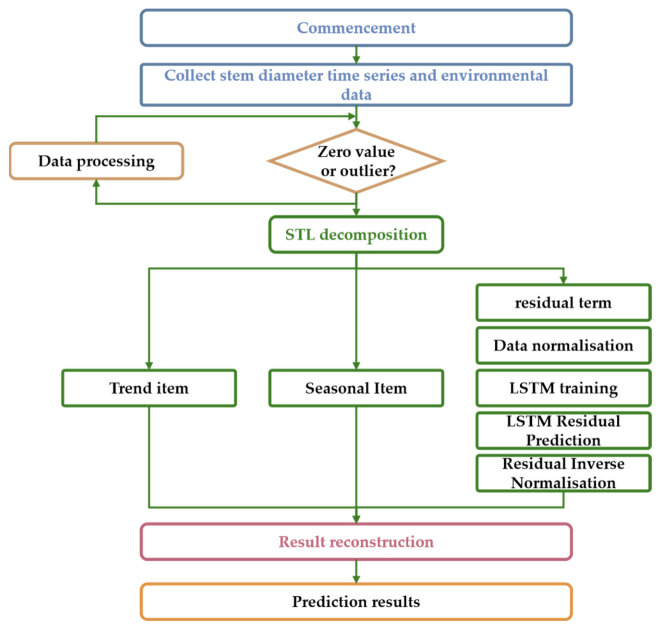
STL-LSTM Model Technology Roadmap.

Prior to model training, a correlation analysis was conducted to quantify the role and degree of influence of these parameters on stem diameter variations (see Figure 6).

**Figure 7:**
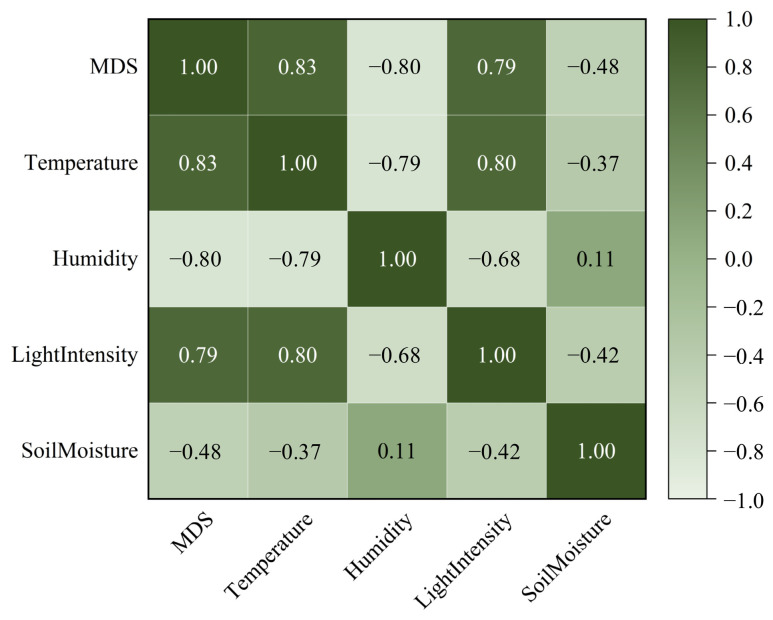
Pearson correlation heatmap between environmental variables and MDS.

The Pearson correlation analysis (Figure 7) reveals that all selected environmental factors significantly influenced the MDS (*p* < 0.05). Specifically, Air Temperature (T), Relative Humidity (H), and Light Intensity (L) exhibited strong correlations with MDS (∣r∣ > 0.79), confirming they are the primary driving forces of diurnal stem dynamics.

Notably, the correlation between T and H was moderate (r = −0.79), avoiding the issue of high multi-collinearity. Furthermore, Soil Moisture (SM) showed a significant moderate negative correlation with MDS (r = −0.48, *p* = 0.031), suggesting that water availability in the rhizosphere provides a critical baseline for plant hydraulic recovery, distinct from the immediate atmospheric demands.

These complex, partially decoupled relationships justify the use of advanced machine learning algorithms to capture non-linear interactions that simple linear models might overlook.

### 3.1. Time Series Decomposition Based on STL

To mitigate the non-stationary interference in the original sequence, the STL algorithm is employed to decompose the stem diameter sequence Y_t_ into three additive components:

(2) $$Y_{t}\;=T_{t}\;+\;S_{t}\;+\;R_{t}$$

Here, T_t_ denotes the trend term (Trend), representing the long-term growth rate of crops; S_t_ denotes the seasonal term (Seasonal), capturing periodic fluctuations driven by circadian rhythms; R_t_ denotes the remainder term (Remainder), reflecting high-frequency random disturbances influenced by environmental fluctuations or physiological regulation.

**Figure 8:**
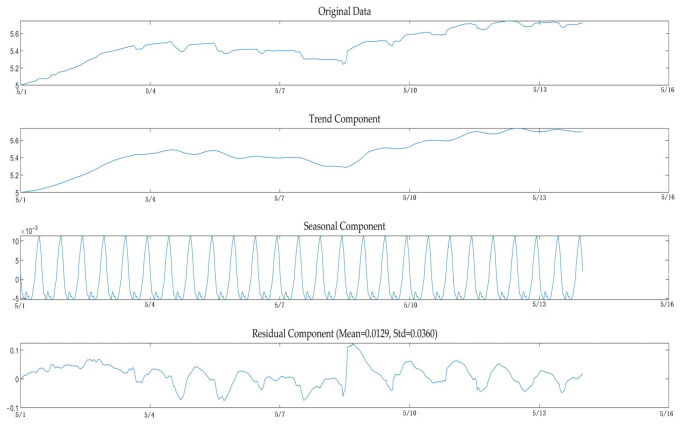
The STL decomposition results of the stem diameter time series.

Based on the data sampling frequency (30 min per measurement), the seasonal cycle parameter was set to 48 to align with the 24-h physiological cycle. The trend smoothing window was set to 336 (equivalent to seven complete cycles) to ensure trend smoothness while preventing leakage of seasonal information. The decomposition results are illustrated in Figure 8.

### 3.2. Weighting Prediction Strategy and Model Construction

In response to the distinct mathematical characteristics of different components, this study adopts a differentiated forecasting strategy.

#### 3.2.1. Deterministic Forecasting of Trend and Seasonal Components

Trend component forecasting: Given the trend component’s high degree of continuity in the short term, a second-order polynomial fitting extrapolation method is employed. A regression model is constructed using historical trend data from the preceding seven days to capture minute variations in growth rates.

Seasonal Component Forecasting: Given the strict cyclical recurrence of seasonal components, the persistence method is employed: S_t+h_ = S_t+h_ − 48. This utilises phase information from the corresponding time point of the preceding day as the forecast value.

#### 3.2.2. Residual Term Correction Based on Multi-Feature Fusion LSTM

**Figure 9:**
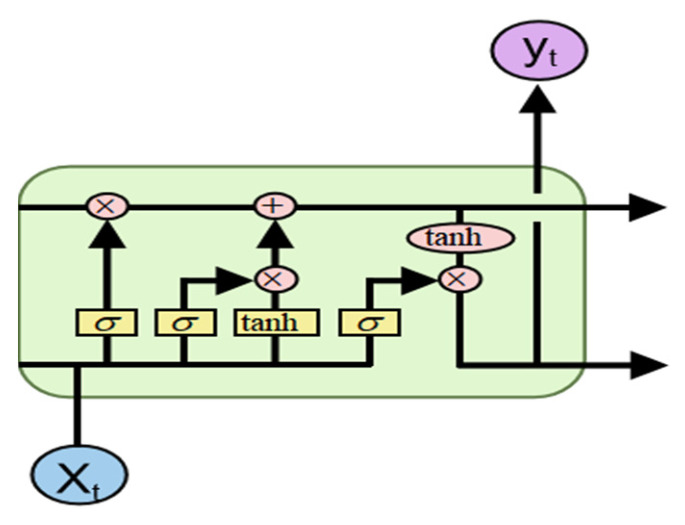
Schematic diagram of heterogeneous LSTM.

The residual term R_t_ encompasses complex nonlinear dynamics and is pivotal for enhancing prediction accuracy. This study constructs a multi-feature input LSTM model to correct residuals, as depicted in Figure 9.

Input feature construction: The input feature vector X comprises: ① the three components of STL over the preceding 24 time steps; ② multidimensional environmental factors (temperature, humidity, light and effective radiation, soil moisture content); ③ Periodic time encoding following sine-cosine transformation.

Network Architecture: A two-layer stacked LSTM structure is employed. The first layer configures 128 hidden units for extracting fundamental temporal features, while the second layer configures 64 units for higher-order abstraction. A Dropout layer (Rate = 0.2) is applied after each layer to mitigate overfitting.

Sliding Window Configuration: The lookback window is set to 24, with a prediction horizon of 48, enabling 24-h advance warning requirements.

### 3.3. Dataset Partitioning and Hyperparameter Optimisation

#### 3.3.1. Time Series Partitioning

To capture a comprehensive range of physiological responses to varying water availability, the dataset for STL-LSTM development was integrated from the preliminary experiment and the Progressive Drought (PD) experiment. The PD experiment, in particular, provided a continuous gradient of water status from field capacity to severe drought, encompassing the water stress levels represented by T1, T2, and T3 in the subsequent validation phase. Considering the temporal correlation of plant growth data, the dataset was partitioned chronologically into a training set (15 March to 1 June), a validation set (2 June to 1 July), and a test set (2 July to 30 July). The validation set constitutes approximately 20% of the total data and is employed to monitor generalisation error during the training process.

#### 3.3.2. Bayesian Hyperparameter Optimisation

Bayesian optimisation algorithms were employed for parameter search on the validation set. All input features underwent Z-score normalisation. The search space and final optimisation results are presented in Table 2.

### 3.4. Hybrid Forecasting Reconstruction and Evaluation Metrics

#### 3.4.1. Result Reconstruction

The final stem diameter prediction value Y_t+h_ is obtained through the linear combination of the component prediction values:

(3) $$Y_{t+h}=T_{t+h}+S_{t+h}+R_{t+h}$$

This strategy combines the robustness of statistical methods in deterministic components with the nonlinear fitting capability of deep learning in random residuals.

#### 3.4.2. Performance Evaluation Metrics

The root mean square error (RMSE), coefficient of determination (R^2^), and mean absolute percentage error (MAPE) are employed as evaluation metrics:

RMSE: Measures the degree of deviation between predicted and actual values, exhibiting high sensitivity to large errors.

(4) $$RMSE=\sqrt{\frac{1}{n}\sum_{i=1}^{n}(y_{i}-{\hat{y}}_{i}{)}^{2}}$$

R^2^: Represents the model’s ability to explain the variability in the original data; the closer it is to 1, the higher the goodness of fit.

(5) $$R^{2}=1-\frac{\sum_{i=1}^{n}(y_{i}-{\hat{y}}_{i}{)}^{2}}{\sum_{i=1}^{n}(y_{i}-\bar{y}{)}^{2}}$$

MAPE: Reflects the relative error level of the forecast results, used to assess the robustness of the model.

(6) $$MAPE=\frac{1}{n}\sum_{i=1}^{n}\left|\frac{y_{i}-{\hat{y}}_{i}}{y_{i}+0.01}\right|\times 100\%$$

## 4. Results

### 4.1. Dynamics and Characteristic Analysis of Tomato Stem Diameter Variations

#### 4.1.1. Diurnal Growth Rhythms and Physiological Responses Under Typical Conditions

**Figure 10:**
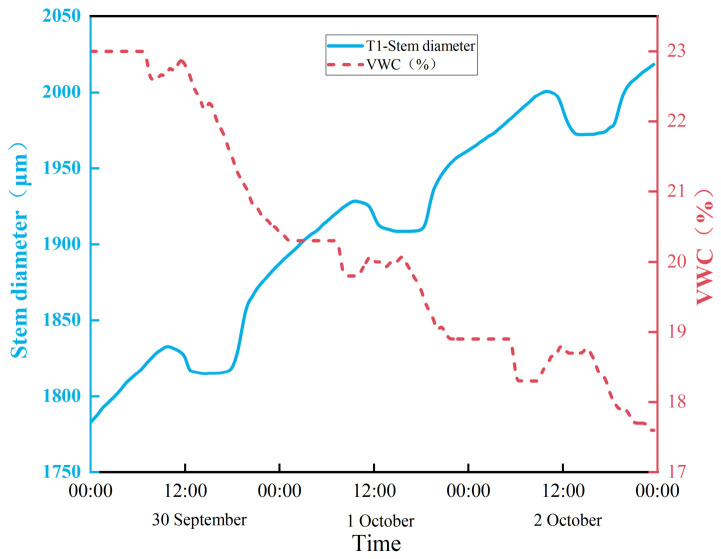
Diagram of daily change in tomato stem diameter.

Figure 10 illustrates the daily dynamic changes in tomato stem diameter from 30 September to 1 October. During this fruiting stage, plant stems had largely matured. The period from 30 September to 3 October featured clear weather, while 4 October was rainy. Research indicates that tomato stem diameter exhibits a 24-h cyclical pattern. Contraction commences around 9:30 am, with diameter decreasing to a minimum between 4:00 pm and 5:00 pm, followed by restorative expansion. This pattern indicates that daytime transpiration causes water loss and stem contraction, while night-time root water uptake replenishes the moisture lost during the day, restoring stem diameter.

As shown in Figure 10, the VWC represents the primary soil-side driver for SDV. We focused on VWC because it directly determines the timing of water stress.

#### 4.1.2. Comparison of Sunny and Cloudy Days

During the tomato fruiting period, data collected over a continuous 30-day period were analyzed, revealing a consistent and significant correlation between environmental driving forces and stem diameter dynamics across the entire duration. To more clearly illustrate the physiological basis and the logical necessity of our proposed decision-making framework, representative data from two days with contrasting weather conditions (2 October: sunny; 4 October: overcast) were selected as typical case studies.

**Figure 11:**
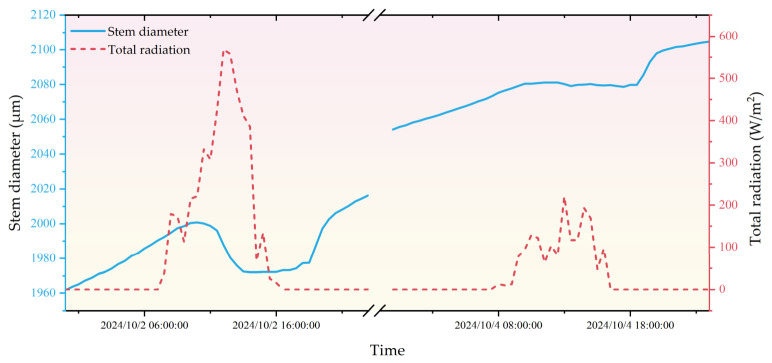
Comparison chart of tomato stem diameter changes on sunny and cloudy days.

As illustrated in Figure 11, solar radiation was selected as the representative atmospheric variable, as it is the dominant factor driving the evapotranspiration process (ET). on 2 October (sunny), the stem diameter exhibited a distinct pattern of diurnal contraction and nocturnal recovery. The diameter reached its daily maximum in the early morning (9:00–10:00) and its minimum in the afternoon (14:00–16:00). This pronounced contraction reflects a transient water imbalance between transpirational loss and root water uptake, driven by high solar radiation and elevated atmospheric evaporative demand. Under such conditions, even when soil moisture is relatively sufficient, the plant may experience a temporary reduction in cell turgor due to the sheer intensity of transpiration. Therefore, this physiological response represents a sensitive adaptation to atmospheric driving forces rather than necessarily indicating a state of chronic or severe water stress. In contrast, on 4 October (overcast), the daily variation was markedly reduced, with a relatively flat curve and a much higher daily minimum diameter. These two days exemplify the typical extremes of weather-driven physiological responses observed throughout the 30-day dataset.

By comparing these contrasting patterns, the graph highlights the sensitivity of MDS to varying weather conditions, which also reveals the inherent limitation of relying solely on MDS for irrigation scheduling. High atmospheric demand on sunny days can trigger ‘false positive’ water stress signals (abnormally high MDS), while low demand on overcast days might mask actual soil water deficits. To overcome these potential misinterpretations, our study integrates soil moisture content (SWC) as a secondary verification. This dual-threshold approach (MDS + SWC) ensures that irrigation is only triggered when both the plant’s physiological response and the root-zone water availability confirm a genuine deficit, thereby providing a more precise and robust assessment for scientifically informed irrigation decisions.

### 4.2. Identification and Quantization of MDS Thresholds Based on Water Stress Induction

In the progressive drought experiments, the maximum daily stem diameter shrinkage (MDS) recorded in the previous day’s treatment group was defined as the potential threshold at which the crop entered water stress. Concurrently, we recorded the corresponding soil water content (SWC) at this point. By repeating multiple rounds of such progressive drought experiments and analysing extensive datasets, this study aims to identify stable and predictive MDS thresholds, along with their corresponding soil water contents, that precede the onset of sustained water stress in crops.

As shown in Table 3, tomato plants in the control group maintained a soil water content (SWC) above 22%, corresponding to a PPV (Percentage of Pore Volume) fraction of >0.85. This moisture level corresponds to approximately 80–90% of field capacity for the specific soil texture used, falling within the ‘readily available water’ (RAW) range where matric potential does not restrict root uptake. Correspondingly, the maximum daily stem diameter shrinkage (MDS) remained within a stable range of 30–70 μm. This physiological stability indicates that the root-zone water availability was sufficient to equilibrate with the daily transpiration demand, preventing significant plant water deficits. No growth arrest was observed throughout this period, thereby validating the MDS range of 30–70 μm as a baseline for tomato plants under non-limiting water conditions.

In the progressive drought treatment group, as soil water content (SWC) gradually decreased, Maximum Daily Shrinkage (MDS) exhibited a marked upward trend. When the PPV declined to approximately 0.62–0.83 (corresponding to 16.2% to 21.5% SWC), MDS values were observed to rise substantially above 84 μm (peaking at 117.17 μm). At this MDS level, all treatment groups had reached a physiologically determined growth arrest state.

Through data analysis of critical points during progressive drought, plants can maintain non-stunted growth when MDS values fall within the 64–75 micrometre range (e.g., MDS 64.35 μm, SWC 20.5%, PPV 0.79; MDS 75.55 μm, SWC 18.0%, PPV 0.69). However, as the MDS value further increases, signs of impending growth cessation begin to emerge around 75 μm (e.g., MDS 79.46 μm, SWC 17.5%, PPV 0.67). Once the MDS surpasses 80 μm, such as at MDS 90.10 μm (SWC 16.5%, PPV 0.63).

Notably, previous studies have highlighted that MDS is a coupled signal influenced both by soil water availability and atmospheric evaporative demand [33] (Ortuño et al., 2010). Extreme environmental factors, such as high vapor pressure deficit (VPD), may cause abnormal increases in MDS values even before soil water reaches a critical deficit, potentially leading to misjudgments of severe crop water deficiency [34,35] (Fernández and Cuevas, 2010; De Swaef et al., 2010). This limitation justifies the necessity of the dual-threshold decision-making approach (MDS-PPV) proposed in this research. By integrating these two indicators (MDS and standardized PPV), our model effectively filters out atmospheric noise, ensuring a more reliable identification of true physiological water stress and preventing premature irrigation.

To provide a statistically robust basis for the early-warning threshold, we applied a Statistical Process Control (SPC) approach to the MDS dataset during the normal growth phase (n = 1152). As shown in Table 4, the baseline MDS was identified at 51.5 ± 21.7 μm. By adopting the upper control limit (UCL) principle (Mean + 1σ), we identified 73.2 μm as the boundary of normal physiological fluctuation. To ensure proactive intervention, a conservative threshold of 70 μm was selected as the irrigation trigger. This threshold is statistically significant, as it captures the deviation exceeding 84% of normal observations, effectively separating the ‘Critical Transition’ phase (SWC ≈ 17.0%, PPV ≈ 0.65) from normal growth. This dual-threshold approach ensures that irrigation is triggered before the onset of irreversible growth restriction.

### 4.3. Performance Evaluation and Error Analysis of the Model

#### 4.3.1. Quantitative Comparison with Benchmark Models

To objectively evaluate the predictive performance of the STL-LSTM model, this study selected the deep learning benchmark model LSTM and the traditional time series model ARIMA as controls. The ARIMA (Auto-Regressive Integrated Moving Average) model is a classical statistical method that relies on the inherent stochastic properties of a time series. Its essence lies in capturing linear relationships and seasonal patterns by combining auto-regressive (AR) processes, differencing (I) to achieve stationarity, and moving averages (MA) of past forecast errors. However, as a univariate linear model, ARIMA often struggles with the high non-linearity and complex noise characteristic of physiological signals like stem thickness.

Table 5 summarises the evaluation metrics of the three models on the validation set. Experimental results demonstrate that the STL-LSTM model outperforms its counterparts across all key metrics, including RMSE, MAPE, and R^2^. While ARIMA provided a basic baseline for the trend, it failed to track the abrupt changes in stem diameter caused by rapid environmental fluctuations. Compared to the standalone LSTM model, which can capture long-term dependencies but may be sensitive to overlapping signals, the STL-LSTM achieves significantly enhanced prediction accuracy. This validates the effectiveness of STL decomposition in partitioning the complex stem thickness signal into distinct components (trend, seasonality, and residuals). By decoupling the underlying growth trend from the high-frequency environmental noise before training, the LSTM is able to learn more stable and representative features, thus leading to superior robustness in forecasting plant water status.

For the critical physiological phase of water stress, this section compares the tracking efficacy of different models regarding stem diameter dynamics. Experimental data indicate that the STL-LSTM model demonstrates optimal performance across all evaluation metrics. Quantitative analysis reveals that during the peak fruiting stage characterised by severe environmental fluctuations, the STL-LSTM model achieves an R^2^ value of 0.9184, representing a 23.5% improvement in prediction accuracy compared to the LSTM model.

**Figure 12:**
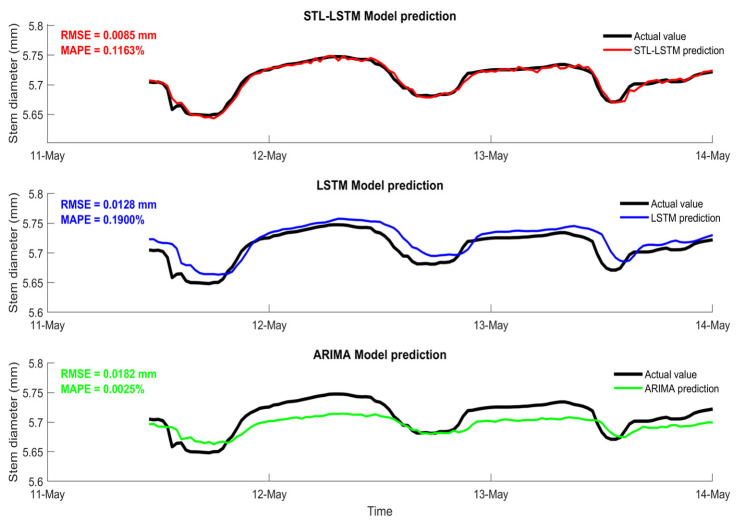
Comparison of predicted and measured stem diameter curves for STL-LSTM, LSTM, and ARIMA models.

As illustrated in Figure 12, the single LSTM model exhibits a time lag in its prediction curve during the water-deficient phase, struggling to respond in real-time to rapid stem diameter contraction. In contrast, the ARIMA model, unable to effectively handle non-stationary time series data, exhibited significantly higher prediction residuals than deep learning models. By performing multi-scale decomposition on the raw sequence, the STL-LSTM model effectively captured the subtle physiological signals during water deficiency, achieving a more reliable digital representation of tomato physiological status.

#### 4.3.2. Overall Fitting Accuracy and Time-Series Feature Analysis

**Figure 13:**
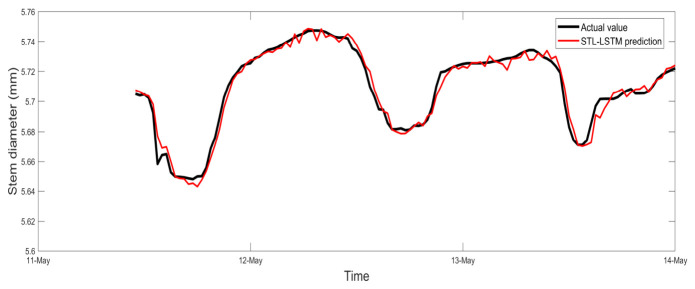
Comparison of predicted and true values.

Figure 13 illustrates the comparison between the observed and predicted values of tomato stem diameter (SD) for the independent validation set. The results demonstrate that the proposed STL-LSTM model exhibits superior predictive fidelity, with the predicted trajectories showing a high degree of agreement with the measured data (R^2^ = 0.9184, RMSE = 0.0085).

Crucially, the model accurately captures the typical “shrinkage-expansion” diurnal physiological rhythm of the tomato stalks. This high level of phase synchronization suggests that the STL decomposition effectively decoupled the multi-scale features of the non-stationary SD signal, thereby reducing the learning complexity for the LSTM network regarding non-linear residuals. The model demonstrates robust amplitude fidelity, not only tracking the long-term growth trend but also precisely fitting the rapid contraction and recovery processes driven by daily environmental fluctuations.

**Figure 14:**
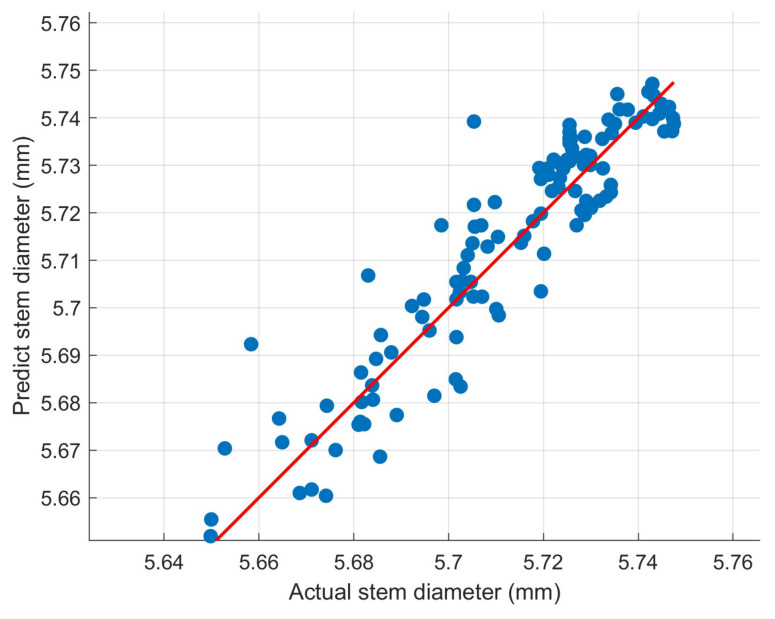
Temporal comparison between measured and predicted stem diameter values for the test period.

Figure 14 illustrates the temporal tracking performance of the STL-LSTM model, demonstrating its ability to capture the diurnal fluctuations of tomato stem diameter. To further quantify the consistency between the observed and predicted values and evaluate the model’s reliability across the entire measurement range, a scatter plot analysis was conducted for the test dataset (Figure 12). As shown in Figure 12, the data points are densely clustered around the 1:1 dashed line, indicating a high degree of correlation. The R^2^ value of 0.9184 and the minimal deviation from the ideal fit further confirm that the model does not exhibit significant systematic bias during both the rapid shrinkage (daytime) and recovery (nighttime) phases.

#### 4.3.3. In-Depth Discussion of Prediction Discrepancies and Error Sources

Despite the high overall accuracy, localized discrepancies—specifically peak clipping and slight phase lags—were observed at extreme points, such as the maximum contraction at noon and the peak expansion at dawn. These deviations can be attributed to the following multifaceted factors:

Non-linear Stochastic Environmental Disturbances: Stem diameter is highly sensitive to micro-environmental fluctuations, such as sudden variations in solar radiation caused by cloud cover. Although the STL decomposition extracts the “Remainder” component, the complex non-linear coupling between high-frequency stochastic noise and plant physiological responses poses a generalization bottleneck for deep learning architectures in handling non-stationary fluctuations.

Biological Hydraulic Inertia and Phase Lag: From a physiological perspective, the response of stem diameter to environmental drivers involves an inherent hydraulic resistance within the plant. During periods of rapid environmental transition, the gating mechanism of the LSTM may struggle to perfectly map the dynamic delays caused by the plant’s internal water potential regulation, resulting in slight temporal shifts or underestimated amplitudes at peak points.

Measurement Uncertainty: Potential sensor drift under extreme humidity/temperature conditions and the inherent growth heterogeneity of individual plants introduce unmodelable noise into the input features, further contributing to the residual error.

In summary, while minor errors persist at extreme fluctuation points, the STL-LSTM model demonstrates sufficient robustness and precision to meet the requirements for real-time crop physiological monitoring and precision irrigation decision-making in greenhouse environments.

## 5. Discussion

This study proposes an irrigation decision-making model integrating physiological indicator prediction with soil moisture status. The core logic of this model lies in leveraging the high sensitivity of crop physiological signals to water deficit, while incorporating soil moisture as a constraint condition. This approach addresses decision biases arising from single-sensor reliance or environmental interference in conventional irrigation strategies.

Experimental results indicate that reliance solely on the maximum daily stem shrinkage (MDS) is susceptible to interference from non-water stress factors such as intense light and high temperatures, generating “false stress” signals. This study introduces a collaborative decision logic of “crop warning-soil confirmation”, setting MDS ≥ 70 μm as the physiological response trigger point, while setting root-zone soil volumetric water content VWC ≤ 17% as the hard constraint for irrigation execution. The advantage of this mechanism lies in its dual capability: it preserves the physiological indicator’s ability to detect early-stage water stress while eliminating false triggers caused by environmental fluctuations through soil moisture status verification. This ensures irrigation decisions are grounded in the plant’s actual water requirement.

Addressing the non-stationary and multi-scale characteristics of stem diameter time series, the STL-LSTM model employed in this study demonstrated superior predictive performance compared to a single LSTM. By decomposing the raw sequence into trend, seasonal, and residual components via STL decomposition, the model effectively reduced the learning complexity of deep neural networks when handling intricate non-linear data. Data comparisons demonstrate that this model can provide a 12-h advance warning window. This temporal gain holds significant practical implications: it shifts irrigation management from traditional ‘post-drought remedial action’ to ‘pre-drought preventative measures’, affording greenhouse automation systems more ample response time.

Furthermore, to address the prospective vision for practical implementation, this framework is designed as a representative-based zonal management system rather than one requiring individual-plant monitoring. In commercial greenhouse production, it is both economically and technically more feasible to deploy sensors on representative ‘sentinel plants’ to capture the collective physiological rhythm of a specific irrigation zone. In this operational model, MDS dynamics serve as the high-sensitivity temporal trigger—answering ‘when’ to irrigate by detecting early-stage physiological demand. By utilizing the plant’s intrinsic physiological response as an ‘integrated biosensor’ for atmospheric demand, this framework reduces the reliance on dense meteorological arrays typically required for calculating reference evapotranspiration (ET0). This ‘sensor-minimalist’ strategy minimizes maintenance costs and operational complexity, providing a more scalable solution for smart agriculture. Meanwhile, soil moisture status serves as the spatial boundary and quantity controller—answering ‘whether’ the biological signal is supported by soil water depletion and ‘how much’ to irrigate. This hierarchical approach—where physiological sentinels dictate timing and soil sensors govern volume—minimizes sensor density requirements while maximizing irrigation precision, providing a scalable solution for smart agriculture.

Although the current model performs well on the validation set, three aspects warrant further investigation. Firstly, the universal applicability of the static thresholds employed in this study (70 μm and 17%) across different growth stages or under extreme climatic conditions remains to be verified. The 3-day evaluation period presented in this study captures the most challenging physiological phase—the onset of net stem shrinkage (DI < 0). Capturing this dynamic shift is the primary objective of our early-warning threshold. However, we acknowledge the reviewer’s concern regarding the limited timeframe. A longer observation period would provide more insights into how the model accounts for age-related physiological changes. To address this, we plan to refine the model in subsequent studies by incorporating a growth-correction factor that adapts to the plant’s phenological development. This will ensure the 70 μm MDS threshold remains robust and reliable throughout the entire cropping season. Developing a dynamic threshold model that adapts to crop growth stages represents a key focus for future research. Secondly, the current decision logic prioritises determining “when to irrigate”; future work should integrate this with evapotranspiration (ET)-based water demand models to simultaneously address “how much to irrigate”. While solar radiation was identified as the dominant driver in this study, future iterations could integrate comprehensive ET0 water demand models to more precisely address the question of “how much to irrigate” during the replenishment phase. Finally, at the algorithmic level, subsequent efforts may explore incorporating meteorological factors as external covariates into multivariate prediction models (e.g., LSTM-X) or evaluating architectures with self-attention mechanisms, such as Transformers, to further enhance model interpretability in complex scenarios.

## 6. Conclusions

To address the limitations of conventional irrigation scheduling in solar greenhouses—specifically the over-reliance on manual experience and single soil moisture indicators—this study analyzed the physiological dynamics of tomato stem diameter and developed a high-precision STL-LSTM predictive model. The main findings are as follows:

(1) Diurnal dynamics and environmental response characteristics of stem diameter.

Tomato stem diameter variations in the arid region of Xinjiang exhibited distinct diurnal rhythms, characterized by nocturnal expansion/recovery and diurnal contraction due to transpiration-induced water deficits. Comparative analysis revealed that the Maximum Daily Shrinkage (MDS) on sunny days was significantly higher than on cloudy days, indicating that solar radiation and high vapor pressure deficit (VPD) are the primary drivers of physiological water fluctuations. The synergistic variation between MDS and Daily Growth (DI) provides a reliable physiological basis for diagnosing the water status of the plant.

(2) Quantization of water stress thresholds and the dual-threshold mechanism.

Through water stress induction experiments, the critical physiological triggers for irrigation were identified. A dual-threshold decision-making mechanism was established by integrating a physiological warning threshold (MDS > 70 μm) with a soil moisture constraint (VWC ≤ 17%). This integrated approach effectively mitigates the risk of false triggers inherent in single-indicator systems under fluctuating environmental conditions, shifting irrigation management from passive replenishment to proactive, plant-centric demand forecasting.

(3) Predictive performance and error characteristics of the STL-LSTM model.

The STL-LSTM model demonstrated superior performance in capturing the multi-scale features of non-stationary physiological sequences through time-series decomposition. During the full-fruiting stage, the model achieved a coefficient of determination (R^2^) of 0.9184, representing an improvement in predictive accuracy of 14.8% and 27.56% over the standalone LSTM and ARIMA models, respectively. Error analysis indicated that while prediction discrepancies primarily originated from the “remainder” component during extreme weather fluctuations, the model remained robust in its overall fitting trend, providing a reliable 24-h forward-looking basis for automated irrigation scheduling.

(4) Prospects for future validation and practical application.

Building upon the physiological triggers and predictive framework established in this study, our subsequent research will focus on the deployment of this system in actual large-scale irrigation scenarios. Future work will aim to further validate and calibrate the proposed dual-threshold mechanism across a wider range of soil textures, crop varieties, and complex field environments, ensuring the long-term reliability and adaptability of the intelligent irrigation decision-making model in practical agricultural production.

## Figures and Tables

**Table 1:** Main technical parameters of the sensors.

Instrument	Model Number	Sensor Name	Measuring Range	Accuracy
Outdoor PH automatic weather station	PH-CJ1	Louvre-type Temperature and humidity	Temperature: −50~+100 °C humidity: 0~100% RH	Temperature: 0.1 °C humidity: 0.1% RH
		Wind Speed Sensor	0~45 m/s	0.1 m/s
		Total Radiation Sensor	0~2000 W/m^2^	1 W/m^2^
		Light intensity sensor	0~200,000 lux	±7%
		CO_2_ concentration sensor	0~5000 ppm	±50 ppm
Indoor wireless agricultural detection system	BNL-GPRS-10G	Total Solar Irradiance Sensor	0~2000 W/m^2^	±1%
		Light intensity sensor	0~200,000 lux	±7%
Wireless Agricultural Meteorological Integrated Monitoring Station	NL-GPRS-I	Tricuspid Soil Temperature and Humidity Sensor	Temperature: −50~+100 °C humidity: 0~100%	Temperature: ±2% FS humidity: ±2.5%
Stem Diameter Sampler	DD-S1	Stem Diameter Sensor	11 mm	0.2 μm (DL18 data logger)
Jingchuang temperature and humidity recorder	GSP-6	Temperature sensor	−40~+85 °C	±0.3 °C
		Humidity sensor	10~99% RH	±3% RH
Weighing equipment	MT202	Platform scale	0.2–30 kg	±1 g

**Table 2:** Space and the final optimisation results.

Hyperparameters	Search Scope	Optimisation Results
Number of LSTM layers	1, 2, 3	2
Number of hidden layer units	32, 64, 128	64
Learning rate	0.001, 0.0001, 0.00001	0.001
Batch size	16, 32, 64	32

**Table 3:** Tomato Water Stress Warning Thresholds and Determination Criteria.

Date	Processing Group	Soil Water Content (swc%)	PPV (Fraction)	Maximum Diameter at Dawn (μm)	Minimum Diameter at Dusk (μm)	MDS (μm)	Physiological Benchmark Assessment (Growth Arrest)
4 May 2025	Control group	26	1.00	5451.73	5417.85	33.93	No
4 May 2025	Processing Group	18.9	0.73	5506.64	5389.47	117.17	Yes
16 May 2025	Control group	25.3	0.97	5872.02	5863.66	8.36	No
16 May 2025	Processing Group	21.5	0.83	5902.91	5818.34	84.57	Yes
25 May 2025	Control group	22.8	0.88	6079.35	6028.39	50.96	No
25 May 2025	Processing Group	16.2	0.62	6106.67	6006.97	99.7	Yes
26 May 2025	Control group	24.5	0.94	6110.12	6065.23	44.89	No
27 May 2025	Processing Group	20.5	0.79	6120.33	6055.98	64.35	No
28 May 2025	Processing Group	18.0	0.69	6125.76	6050.21	75.55	No
29 May 2025	Processing Group	17.5	0.67	6130.45	6050.99	79.46	About to come to a standstill
30 May 2025	Processing Group	16.5	0.63	6135.10	6045.00	90.10	Yes
1 June 2025	Processing Group	19.2	0.74	5990.25	5925.80	64.45	No
2 June 2025	Processing Group	17.0	0.65	6000.10	5930.50	69.60	No
3 June 2025	Processing Group	15.8	0.61	6010.55	5935.10	75.45	About to come to a standstill

**Table 4:** Statistical characteristics of physiological indicators under different moisture conditions.

Physiological State	Sample Duration (d)	Total Data Points (n)	SWC (%) (Mean ± SD)	PPV (Fraction)	MDS (μm) (Mean ± SD)
Normal Growth	8	1152	21.7 ± 3.2	0.83 ± 0.12	51.5 ± 21.7
Water Stress	6	864	17.7 ± 2.1	0.68 ± 0.08	91.1 ± 15.2
Critical Transition	1	144	17.0	0.65	69.6

**Table 5:** Comparison of performance metrics for different prediction models.

Model	RMSE	MAPE	R^2^
LSTM	0.0128	0.1900%	0.8000
ARIMA	0.0182	0.0025%	0.7200
STL-LSTM	0.0085	0.1163%	0.9184

## Data Availability

The data presented in this study are available on request from the corresponding author.
